# Survival Outcomes Following Yttrium-90 and Holmium-166 Transarterial Radioembolization for Hepatocellular Carcinoma

**DOI:** 10.3390/cancers18132039

**Published:** 2026-06-24

**Authors:** Dávid Ádám Korda, Dénes Balázs Horváthy, Sándor Czibor, Domonkos Nádasdy-Horváth, Petra Sólymos, Oszkár Háhn, Bálint Tegze, Klára Werling, Attila Jakó, Szabolcs Takács, Pál Ákos Deák, András Bibok

**Affiliations:** 1 Department of Interventional Radiology, Heart and Vascular Centre, Semmelweis University, Határőr út 18, H-1122 Budapest, Hungary; horvathy.denes@semmelweis.hu (D.B.H.); jako.attila@stud.semmelweis.hu (A.J.); deak.pal.akos@semmelweis.hu (P.Á.D.); bibok.andras@semmelweis.hu (A.B.); 2 Department of Nuclear Medicine, Medical Imaging Centre, Semmelweis University, Korányi Sándor utca 2, H-1083 Budapest, Hungary; czibor.sandor@semmelweis.hu (S.C.); nadasdy.horvath.domonkos@semmelweis.hu (D.N.-H.); 3 Department of Radiology, Medical Imaging Centre, Semmelweis University, Korányi Sándor utca 2, H-1083 Budapest, Hungary; solymos.petra@semmelweis.hu; 4 Department of Surgery, Transplantation and Gastroenterology, Semmelweis University, Üllői út 78, H-1082 Budapest, Hungary; hahn.oszkar@semmelweis.hu (O.H.); werling.klara@semmelweis.hu (K.W.); 5 Department of Internal Medicine and Haematology, Semmelweis University, Szentkirályi u. 46, H-1088 Budapest, Hungary; tegze.balint@semmelweis.hu; 6 Faculty of Humanities, Károli Gáspár University of the Reformed Church in Hungary, Kálvin tér 9, H-1091 Budapest, Hungary; takacs.szabolcs@kre.hu

**Keywords:** transarterial radioembolization, hepatocellular carcinoma, Yttrium-90, Holmium-166, overall survival, progression-free survival

## Abstract

**Background/Objectives**: Transarterial radioembolization (TARE) plays an increasing role in the treatment of hepatocellular carcinoma (HCC). Several isotope platforms have been introduced; however, comparative data remain limited. We aimed to compare outcomes following Holmium-166 (Ho-166) and Yttrium-90 (Y-90) TARE in HCC patients. **Methods**: We retrospectively analyzed a prospectively maintained cohort of HCC patients treated with Ho-166 or Y-90 TARE between 2022 and 2024. Tumor burden and treatment response were assessed using contrast-enhanced CT or MRI. Overall survival (OS) and progression-free survival (PFS) were estimated using the Kaplan–Meier method and further evaluated using multivariable Cox regression analyses adjusted for baseline confounders. **Results**: A total of 73 patients were analyzed, including 39 treated with Y-90 and 34 with Ho-166 TARE. Median follow-up duration was 20.9 months. No significant differences in OS or PFS were observed between treatment groups. Response outcomes, including objective response rate, complete response rate, and duration of response, were also comparable between groups. In the overall cohort, median OS was not reached in Barcelona Clinic Liver Cancer (BCLC) A or B patients and was 12.9 months in BCLC C patients. Median PFS was 20.1, 11.7, and 6.1 months in BCLC A, B, and C patients, respectively. **Conclusions**: Ho-166 and Y-90 TARE achieved favorable outcomes across different BCLC stages without significant differences in survival, treatment response, or duration of response between the two platforms. These findings further support the role of TARE in the management of patients with HCC.

## Simple Summary

Transarterial radioembolization is increasingly used in patients with hepatocellular carcinoma who are not candidates for surgery or local ablative therapies. Different isotope platforms are available for this treatment, yet direct comparisons remain limited. In this study, we compared oncological outcomes between Holmium-166 and Yttrium-90 radioembolization in patients with hepatocellular carcinoma. Despite differences in imaging and treatment-planning characteristics between the two platforms, no statistically significant differences in survival outcomes, treatment response, or duration of response were observed between treatment groups. Both approaches were associated with favorable oncological outcomes across different BCLC stages.

## 1. Introduction

Interventional radiology has played a central role in the management of hepatocellular carcinoma (HCC) for several decades. Various interventional treatment options are available across nearly all stages of the disease, many of which are included in current clinical guidelines and, in selected cases, are recommended as first-line therapies [1,2,3].

Over the past decade, the role of transarterial radioembolization (TARE) in HCC has evolved substantially. Recent updates of the Barcelona Clinic Liver Cancer (BCLC) staging system have incorporated it as a treatment option in various clinical scenarios, reflecting its growing clinical acceptance and the expanding evidence base supporting its use [4].

TARE is most commonly considered in patients with solitary lesions not amenable to surgery or ablation, in those unsuitable for or refractory to transarterial chemoembolization (TACE), in patients with unilobar disease as a bridge to surgery, and in those with portal vein tumor thrombosis (PVTT) [5,6,7,8].

Despite its increasing use across a range of clinical scenarios, variability in treatment outcomes following TARE has been observed, which may in part be related to technical differences between treatment approaches [9,10]. Two Yttrium-90 (Y-90) radioembolization platforms have traditionally been available, while more recently, Holmium-166 (Ho-166) radioembolization has been introduced in selected centers [11,12,13,14,15]. Although both Ho-166 and Y-90 TARE are based on the selective intra-arterial delivery of radioactive microspheres, the two platforms differ in several technical and dosimetric aspects. One of the key technical features of Ho-166 radioembolization is the use of identical scout and therapeutic particles [16,17,18]. In contrast, treatment planning for Y-90 TARE is typically based on Technetium-99m macroaggregated albumin (Tc-99m MAA). Consequently, the distribution of Tc-99m MAA may not fully reflect the subsequent distribution of therapeutic microspheres during treatment [18]. In addition, Ho-166 microspheres are directly visible on MRI, allowing MRI-based post-treatment imaging and dosimetric assessment [16,17]. The physical characteristics of Ho-166 microspheres are intermediate between the two established Y-90 platforms, potentially resulting in a relatively low embolic effect and more homogeneous microsphere distribution [19,20]. Despite these technical and dosimetric differences, direct comparative data on oncologic outcomes between Y-90- and Ho-166-based radioembolization remain limited. In this context, we aimed to compare oncologic outcomes following TARE using Y-90 glass microspheres and Ho-166-labeled microspheres.

## 2. Materials and Methods

### 2.1. Study Population and Treatment Procedure

We performed a retrospective analysis of a prospectively maintained cohort of patients treated with TARE for HCC. A total of 73 consecutive patients were included between August 2022 and May 2025. Of these, 34 patients underwent Ho-166-based TARE and 39 received Y-90 glass microsphere treatment. Eligible patients had histologically confirmed HCC not amenable to surgical resection or thermal ablation and were selected for TARE by a multidisciplinary tumor board. Additional inclusion criteria included compensated liver disease (Child-Pugh class A or B), Eastern Cooperative Oncology Group (ECOG) performance status 0–1, estimated life expectancy ≥12 weeks, and written informed consent. Exclusion criteria included extrahepatic metastatic disease, decompensated liver disease, uncontrolled infection, pregnancy or breastfeeding, severe contrast media allergy not manageable with premedication, and the presence of another active malignancy requiring systemic oncologic treatment. The choice between Y-90 and Ho-166 radioembolization was non-randomized and was influenced by microsphere availability, multidisciplinary team decision-making, and evolving institutional practice patterns over time.

### 2.2. Treatment Planning and Radioembolization Procedure

Prior to treatment, all patients underwent catheter angiography to identify tumor-feeding arteries. Cone-beam computed tomography (CBCT) acquisitions were performed from all intended treatment positions. Depending on procedural logistics, either during the angiographic session or during a separate planning procedure, Tc-99m MAA was administered prior to Y-90 treatment, whereas a Ho-166 scout dose was administered prior to Ho-166-based radioembolization. Single-photon emission computed tomography combined with computed tomography (SPECT/CT) was performed to assess intrahepatic isotope distribution and lung shunting. Preprocedural imaging studies, SPECT/CT datasets, and CBCT acquisitions obtained from different treatment positions in multi-position treatments were imported into dedicated vendor-specific software platforms (Q-Suite™ software version 2.1, Quirem Medical B.V., Deventer, The Netherlands and Simplicit90Y™ software version 2.8, Mirada Medical Ltd., Oxford, UK). Personalized dosimetric planning was subsequently performed to determine the activity required for safe and effective treatment. Post-treatment activity distribution was assessed using positron emission tomography combined with computed tomography (PET/CT) after Y-90 radioembolization and magnetic resonance imaging (MRI) alongside SPECT/CT after Ho-166 treatment. Baseline demographic, clinical, laboratory, and imaging data were collected prior to treatment. Tumor burden was assessed on baseline contrast-enhanced CT or MRI according to modified Response Evaluation Criteria in Solid Tumors (mRECIST). BCLC stage and Child-Pugh class were determined prior to treatment initiation.

### 2.3. Outcome Assessment and Follow-Up

Follow-up consisted of clinical visits and contrast-enhanced CT or MRI examinations performed at 3-month intervals. Imaging studies were reviewed by an independent diagnostic radiologist together with an interventional radiologist experienced in TARE procedures. Treatment response was assessed according to mRECIST. The best therapeutic response corresponded to the best recorded response during follow-up according to mRECIST criteria. Objective response rate (ORR) was defined as the proportion of patients achieving complete response (CR) or partial response (PR), while complete response rate (CRR) was defined as the proportion achieving CR. Duration of response (DoR) was defined as the time from first documented objective response (CR or PR) until radiological progression or death from any cause. Patients who underwent liver transplantation or surgical resection before progression were censored at the time of intervention. Overall survival (OS) was defined as the time from TARE treatment to death from any cause or last follow-up, while progression-free survival (PFS) was defined as the time from treatment to radiological progression or death. In addition, laboratory examinations were performed 2–4 weeks after radioembolization to evaluate for liver dysfunction or hepatic decompensation. Subsequent follow-up was individualized based on the recommendations of the treating hepatologist.

### 2.4. Statistical Analysis

Statistical analyses were performed using STATA version 19.5 statistical software (StataCorp LLC, College Station, TX, USA). Continuous variables were expressed as mean ± standard deviation or median with interquartile range (IQR), as appropriate, while categorical variables were summarized as frequencies and percentages. Baseline characteristics between treatment groups were compared using Student’s *t*-test, Mann–Whitney U test, Fisher’s exact test, or chi-square test, as appropriate. OS and PFS were estimated using the Kaplan–Meier method and compared between treatment groups using the log-rank test. Univariable and multivariable Cox proportional hazards regression analyses were performed to evaluate associations between treatment type and survival outcomes. Results of Cox regression analyses were reported as hazard ratios (HRs) with corresponding 95% confidence intervals (CIs). Multivariable models were adjusted for BCLC stage and age, selected based on clinical relevance and baseline imbalance. In addition, BCLC-stratified Cox regression analyses were performed. Sensitivity analyses were conducted using alternative multivariable models including baseline sum of diameters (SOD) of target lesions measured according to mRECIST criteria, PVTT, and age. Median survival outcomes according to BCLC stage were additionally estimated using the Kaplan–Meier method. All statistical tests were two-sided, and a *p*-value < 0.05 was considered statistically significant. No substantial missing baseline or follow-up data were present. The anonymized study dataset is provided as Dataset S1 in the Supplementary Materials.

### 2.5. Ethics

The study was conducted in accordance with the Declaration of Helsinki and approved by the Regional and Institutional Committee of Science and Research Ethics of Semmelweis University (SE RKEB No. 27/2026).

## 3. Results

### 3.1. Baseline Characteristics

The study population consisted predominantly of patients with BCLC A disease and preserved hepatic function. Overall, 48 patients (66%) had BCLC A disease, 14 (19%) had BCLC B disease, and 11 (15%) had BCLC C disease. In all patients with BCLC C disease, the reason for advanced-stage classification was PVTT. Child-Pugh class A liver function was present in 67 patients (92%), while the remaining 6 patients (8%) had Child-Pugh class B liver function. The most common underlying liver disease etiologies were viral hepatitis and alcohol-related liver disease (ARLD), while metabolic dysfunction-associated steatotic liver disease (MASLD) was also frequently present. Baseline demographic, clinical, and tumor characteristics were comparable between treatment groups, with the exception of age. Patients treated with Y-90 were significantly older than those treated with Ho-166 (72.4 ± 6.9 vs. 67.4 ± 6.6 years, *p* = 0.003) (Table 1). Median follow-up duration estimated using the reverse Kaplan–Meier method was 20.9 months. Dosimetric parameters are summarized in Supplementary Table S1.

### 3.2. OS Comparison Between Treatment Groups

**Figure 1:**
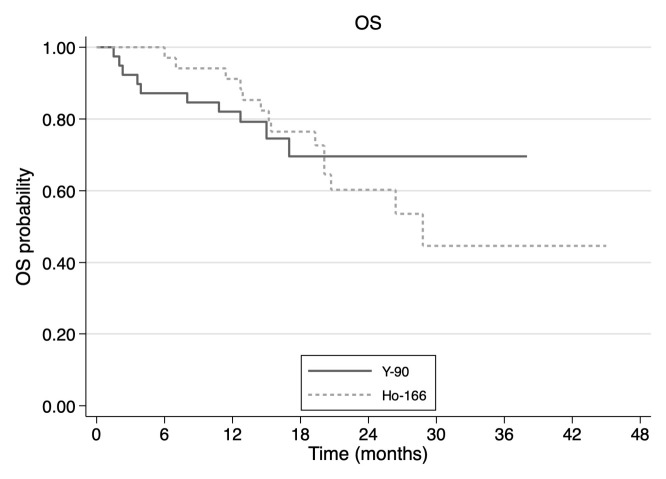
Kaplan–Meier curves of overall survival (OS) following transarterial radioembolization with Yttrium-90 (Y-90) and Holmium-166 (Ho-166) microspheres in patients with hepatocellular carcinoma. Differences between treatment groups were evaluated using the log-rank test.

No significant difference in OS was identified between treatment groups in Kaplan–Meier analysis (log-rank *p* = 0.700, Figure 1) or in univariable Cox regression analysis (HR 1.17, 95% CI 0.52–2.66, *p* = 0.701).

Similarly, multivariable Cox regression adjusted for BCLC stage and age demonstrated no association between treatment type and OS (HR 1.05, 95% CI 0.44–2.52, *p* = 0.913). BCLC C disease was independently associated with significantly worse OS compared with BCLC A disease (HR 4.91, 95% CI 1.78–13.50, *p* = 0.002).

In the BCLC-stratified Cox regression model adjusted for age, treatment type remained unassociated with OS (HR 1.01, 95% CI 0.41–2.47, *p* = 0.988). Sensitivity analysis including baseline SOD, PVTT, and age yielded comparable findings (HR 1.18, 95% CI 0.49–2.85, *p* = 0.711), while the presence of PVTT remained independently associated with significantly worse OS (HR 3.93, 95% CI 1.54–10.02, *p* = 0.004).

### 3.3. PFS Comparison Between Treatment Groups

**Figure 2:**
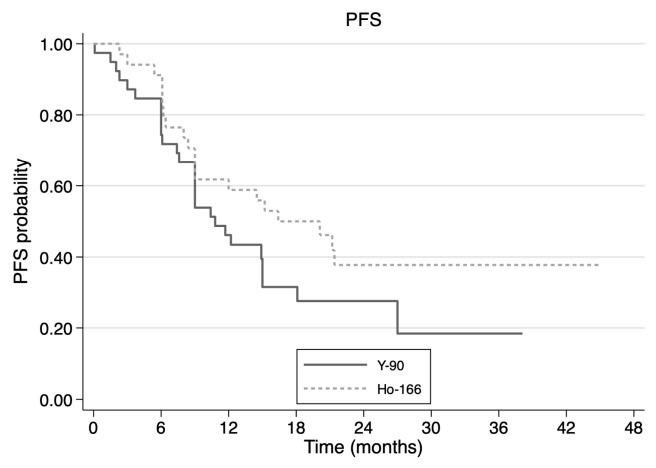
Kaplan–Meier curves of progression-free survival (PFS) following transarterial radioembolization with Yttrium-90 (Y-90) and Holmium-166 (Ho-166) microspheres in patients with hepatocellular carcinoma. Differences between treatment groups were evaluated using the log-rank test.

Kaplan–Meier analysis demonstrated no statistically significant difference in PFS between treatment groups (log-rank *p* = 0.118, Figure 2). However, univariable Cox regression analysis showed a consistent trend toward prolonged PFS in patients treated with Ho-166 microspheres compared with Y-90 treatment (HR 0.63, 95% CI 0.35–1.14, *p* = 0.127).

After adjustment for BCLC stage and age, the association remained directionally similar but did not reach statistical significance (HR 0.59, 95% CI 0.32–1.08, *p* = 0.089). BCLC C disease was independently associated with significantly worse PFS compared with BCLC A disease (HR 3.20, 95% CI 1.51–6.77, *p* = 0.002), whereas age was not associated with PFS.

In the BCLC-stratified Cox regression model adjusted for age, a similar non-significant trend favoring Ho-166 treatment was seen (HR 0.60, 95% CI 0.32–1.11, *p* = 0.103).

Sensitivity analysis including baseline SOD, PVTT, and age demonstrated comparable findings (HR 0.61, 95% CI 0.32–1.14, *p* = 0.120). In this model, the presence of PVTT was independently associated with significantly shorter PFS (HR 2.72, 95% CI 1.29–5.74, *p* = 0.009).

### 3.4. Treatment Response and Duration of Response

Treatment response was comparable between treatment groups. Objective response was achieved in 32/39 (82.1%) patients treated with Y-90 and 28/34 (82.4%) patients treated with Ho-166 microspheres (*p* = 1.000). Complete response was observed in 24/39 (61.5%) and 19/34 (55.9%) patients, respectively (*p* = 0.642) (Table 2).

**Figure 3:**
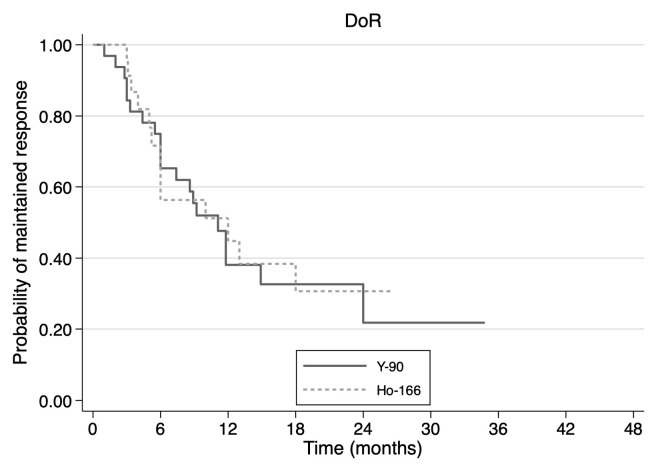
Kaplan–Meier curves of duration of response (DoR) among responding patients following transarterial radioembolization with Yttrium-90 (Y-90) and Holmium-166 (Ho-166) microspheres. Differences between treatment groups were evaluated using the log-rank test.

Among responding patients, no significant difference in duration of response was observed between treatment groups in Kaplan–Meier analysis (log-rank *p* = 0.813, Figure 3). Similarly, univariable Cox regression demonstrated no association between treatment type and duration of response (HR 0.92, 95% CI 0.46–1.86, *p* = 0.816). After adjustment for BCLC stage and age, treatment type remained unassociated with duration of response (HR 0.90, 95% CI 0.42–1.95, *p* = 0.791). Sensitivity analysis incorporating baseline SOD, PVTT, and age yielded comparable findings (HR 0.90, 95% CI 0.41–1.98, *p* = 0.801).

### 3.5. BCLC-Stratified Survival Outcomes of the Entire Cohort

Survival outcomes differed substantially across BCLC stages. Median PFS was 20.1 months in BCLC A patients, 11.7 months in BCLC B patients, and 6.1 months in BCLC C patients. Median OS was not reached in BCLC A and B patients, while BCLC C patients had a median OS of 12.9 months (Table 3).

### 3.6. Subsequent Treatments Following TARE

Subsequent treatments following TARE were performed in 22/39 (56.4%) patients in the Y-90 cohort and 27/34 (79.4%) patients in the Ho-166 cohort. Curative-intent interventions, including liver transplantation and surgical resection, were performed in selected patients following favorable tumor response or successful downstaging, while other locoregional and systemic therapies were primarily administered after disease progression. Subsequent treatments are summarized in Table 4.

### 3.7. Complications

Treatment-related adverse events are summarized in Table 5. Most adverse events were mild and consistent with post-radioembolization syndrome, including abdominal pain, fever/subfebrility, and nausea. Additional minor adverse events included transient transaminase elevation, access-site hematoma, and syncope. No cases of gastrointestinal ulceration or radiation pneumonitis were identified. Two major complications were observed in the study population (major complication rate: 2.7%). Both were classified as grade 6 according to the modified Cardiovascular and Interventional Radiological Society of Europe (CIRSE) classification system and occurred following Y-90 TARE treatment [21]. One patient developed a biliary stricture requiring percutaneous transhepatic drainage. Despite biliary decompression, the clinical course was subsequently complicated by cholangitis, and the patient ultimately died from sepsis two months after treatment. Another patient with underlying Child–Pugh B cirrhosis underwent treatment of two lesions located in separate hepatic lobes. Both lesions were treated from superselective catheter positions using conservative dosimetry; however, progressive hepatic decompensation ensued, resulting in liver failure and death three months after treatment.

## 4. Discussion

In the present study, TARE was associated with favorable safety and clinically meaningful oncological outcomes across BCLC stages. No significant differences were observed between Ho-166 and Y-90 treatment with regard to OS, ORR, CRR, or DoR. Although patients treated with Ho-166 microspheres showed a consistent trend toward prolonged PFS, this association did not reach statistical significance. Survival outcomes were strongly associated with tumor stage, with substantially longer PFS and OS in patients with earlier-stage disease.

Treatment-related toxicity was generally limited within the present cohort, with one severe biliary complication and one case of hepatic decompensation after TARE. Clinically significant biliary complications requiring intervention are relatively uncommon following TARE; however, centrally located tumors adjacent to the biliary confluence may carry an increased risk of biliary injury [22]. Hepatic decompensation is a recognized complication of TARE in cirrhotic patients, particularly following bilobar treatment and in patients with impaired baseline liver function [23,24]. Superselective treatment approaches may reduce the risk of non-target parenchymal injury.

Patients with BCLC A disease achieved prolonged disease control, with a median PFS of 20.1 months, while median OS was not reached during follow-up. Importantly, these patients were not candidates for curative-intent therapies, including surgical resection or thermal ablation, due to underlying cirrhosis, unfavorable tumor location, or tumor size. Earlier selective radioembolization studies already demonstrated promising survival outcomes in patients with unresectable early-stage HCC [25]. With the increasing adoption of personalized dosimetry and modern selective treatment approaches, recent studies have reported even more favorable outcomes. In the LEGACY study, selective Y-90 radioembolization in patients with solitary unresectable HCC resulted in a 3-year overall survival exceeding 85% [7]. Similarly, the DOSISPHERE-01 trial demonstrated significantly improved survival following personalized dosimetry compared with standard dosimetry approaches [26].

Clinical outcomes remained encouraging in patients with BCLC B disease, with a median PFS of 11.7 months, while median OS was not reached during follow-up. TACE remains the standard locoregional treatment for intermediate-stage HCC, and several studies have reported broadly comparable OS following TARE and TACE. Importantly, TARE has consistently been associated with longer time-to-progression, reduced postembolization syndrome, and improved quality of life [27]. In the randomized PREMIERE trial, TARE was additionally associated with fewer retreatment procedures compared with TACE, likely reflecting more durable tumor control following a single treatment session [28]. Careful patient selection remains essential in the BCLC B setting, as TARE is generally most suitable in patients with limited intrahepatic tumor burden and sufficient preserved liver parenchyma.

All patients with BCLC C disease in the present cohort had PVTT, a condition associated with particularly poor prognosis and limited treatment options. The therapeutic landscape of advanced HCC has evolved in recent years with the introduction of immune checkpoint inhibitor-based systemic therapies. In contemporary real-world cohorts of patients with PVTT treated with atezolizumab plus bevacizumab, median OS has generally been reported around 10–12 months, with median PFS of approximately 4–8 months, although outcomes vary substantially depending on tumor burden, extent of vascular invasion, and underlying liver function [29,30]. Previous TARE cohorts in patients with PVTT have similarly reported median OS ranging between approximately 10 and 15 months [31,32,33]. In this setting, TARE may represent a valuable complementary locoregional treatment modality due to its ability to achieve selective intrahepatic tumor control while preserving uninvolved liver parenchyma. Although prospective data regarding the combination of TARE and immunotherapy remain limited, early retrospective studies suggest that integrating locoregional and systemic therapies is feasible and appears to have an acceptable safety profile [34]. The outcomes in our BCLC C cohort further support continued investigation of these combined treatment approaches in selected patients with PVTT.

In patients with BCLC A and B disease, effective tumor control following TARE may additionally facilitate subsequent curative-intent interventions, including liver transplantation or surgical resection [6,35,36], as reflected in the present cohort. Furthermore, emerging evidence suggests that in highly selected patients, successful downstaging following TARE may even enable liver transplantation in the setting of PVTT [36].

Direct comparisons of oncological outcomes between Ho-166 and Y-90 radioembolization remain limited. Within this cohort, Ho-166 TARE showed a numerical trend toward improved PFS; however, no statistically significant differences in OS or PFS were observed between treatment groups in either univariable or multivariable analyses. Current literature has not demonstrated clear oncological superiority of either isotope platform, with most reported differences primarily involving technical and dosimetric characteristics rather than survival outcomes [11,12,16,17]. Similarly, treatment response outcomes were comparable between treatment groups. No significant differences were observed in ORR, CRR, or DoR, further supporting the absence of clear evidence for oncological superiority of either isotope platform within the present cohort. Importantly, interpretation of the observed survival trends in our cohort should be performed cautiously. A greater proportion of patients in the Ho-166 group underwent subsequent curative-intent interventions, including liver transplantation and surgical resection, which may have contributed to the observed differences in oncological outcomes between treatment groups.

Our study has several limitations. First, its retrospective design and relatively limited sample size may have introduced selection bias and limited statistical power. As a result, the study may have been underpowered to detect more modest differences in progression-free survival between treatment groups. Although patient follow-up was performed within a prospectively maintained cohort, treatment allocation was non-randomized and was influenced by microsphere availability, multidisciplinary decision-making, and evolving institutional practice patterns over time. To mitigate potential confounding, multivariable Cox regression analyses were performed adjusting for clinically relevant baseline characteristics, including BCLC stage, portal vein tumor thrombosis, age, and baseline tumor burden. Nevertheless, residual confounding cannot be excluded, particularly given the significant age difference between treatment groups. In addition, post-TARE therapies differed between groups and may have influenced long-term oncological outcomes. Finally, Ho-166 microspheres are currently not commercially available, which limits the direct clinical applicability of our findings. Nevertheless, evaluation of outcomes achieved with the Ho-166 platform remains relevant, as it represented a valuable adjunct in the treatment of HCC and contributes to the limited body of evidence available regarding different radioembolization platforms. Despite these limitations, to our knowledge, this study represents one of the largest comparative cohorts evaluating oncological outcomes following Ho-166 radioembolization in patients with HCC.

## 5. Conclusions

Both Ho-166 and Y-90 TARE yielded favorable oncological outcomes across different BCLC stages in patients with HCC. Despite important differences in the physical and imaging characteristics of the two microspheres, survival outcomes, treatment response, and duration of response were comparable between treatment groups. These findings further support the role of TARE as an effective treatment option for patients with HCC.

## Figures and Tables

**Table 1:** Baseline demographic, clinical, and tumor characteristics of the study population stratified by treatment group. Continuous variables are presented as mean ± standard deviation or median (interquartile range [IQR]), as appropriate. Categorical variables are presented as counts and percentages. Abbreviations: SD, standard deviation; IQR, interquartile range; SOD, sum of diameters; PVTT, portal vein tumor thrombosis; ARLD, alcohol-related liver disease; MASLD, metabolic dysfunction-associated steatotic liver disease; BCLC, Barcelona Clinic Liver Cancer.

Variable	Y-90 (*n* = 39)	Ho-166 (*n* = 34)	*p*-Value
**Age, years, mean ± SD**	72.4 ± 6.9	67.4 ± 6.6	**0.003**
**Male sex, ** ***n*** ** (%)**	22 (56%)	25 (74%)	0.148
**Child–Pugh B, ** ***n*** ** (%)**	3 (8%)	3 (9%)	1.000
**Albumin, g/L, mean ± SD**	40.8 ± 4.7	40.8 ± 5.3	0.945
**Bilirubin, µmol/L, median (IQR)**	14.4 (10.2–21.3)	17.15 (12.5–25.4)	0.176
**Baseline SOD, median (IQR)**	5.0 (4.0–9.0)	5.35 (4.0–8.5)	0.488
**PVTT, ** ***n*** ** (%)**	5 (13%)	6 (18%)	0.745
**Cirrhosis etiology, ** ***n*** ** (%)**			0.714
Viral hepatitis	16 (41%)	15 (44%)	
ARLD	15 (38%)	9 (26.5%)	
MASLD	7 (18%)	9 (26.5%)	
Other	1 (3%)	1 (3%)	
**BCLC stage, ** ***n*** ** (%)**			0.884
BCLC A	26 (67%)	22 (65%)	
BCLC B	8 (21%)	6 (18%)	
BCLC C	5 (13%)	6 (18%)	
**Prior treatment, ** ***n*** ** (%)**	10 (26%)	6 (18%)	0.572

**Table 2:** Treatment response outcomes following transarterial radioembolization with Y-90 and Ho-166 microspheres in patients with hepatocellular carcinoma. Response assessment was performed according to mRECIST criteria. Abbreviations: ORR, objective response rate; CRR, complete response rate.

Outcome	Y-90 (*n* = 39)	Ho-166 (*n* = 34)	*p*-Value
ORR, *n* (%)	32 (82.1%)	28 (82.4%)	1.000
CRR, *n* (%)	24 (61.5%)	19 (55.9%)	0.642

**Table 3:** Survival outcomes according to the BCLC stage. Median progression-free survival (PFS) and overall survival (OS) were estimated using the Kaplan–Meier method. Median OS was considered not reached when the Kaplan–Meier survival estimate did not decline below 50% during follow-up. Abbreviations: BCLC, Barcelona Clinic Liver Cancer; OS, overall survival; PFS, progression-free survival.

BCLC Stage	Median PFS (Months)	Median OS (Months)
BCLC A	20.1	not reached
BCLC B	11.7	not reached
BCLC C	6.1	12.9

**Table 4:** Subsequent treatments following TARE in the Y-90 and Ho-166 cohorts. Patients may have received multiple subsequent treatments; therefore, individual patients may be represented in more than one category. Abbreviations: Ho-166, Holmium-166; TACE, transarterial chemoembolization; TARE, transarterial radioembolization; Y-90, Yttrium-90.

Subsequent Treatments	Y-90 (*n* = 39)	Ho-166 (*n* = 34)
Liver transplantation	1	9
Surgical resection	1	3
Thermal ablation	3	2
TACE	4	8
Repeat TARE	1	0
Systemic Therapy	14	9

**Table 5:** Complications following transarterial radioembolization with Yttrium-90 (Y-90) and Holmium-166 (Ho-166) microspheres in patients with hepatocellular carcinoma. Adverse events were graded according to the modified Cardiovascular and Interventional Radiological Society of Europe (CIRSE) classification system. Data are presented as *n* (%). Patients may have experienced more than one adverse event and may therefore be represented in multiple categories.

Complication	mCIRSE Grade	Y-90 (*n* = 39)	Ho-166 (*n* = 34)
Abdominal pain	1–2	3 (7.7%)	5 (14.7%)
Fever/subfebrility	1–2	1 (2.6%)	1 (2.9%)
Nausea	1a	1 (2.6%)	0
Access-site hematoma	1a	1 (2.6%)	0
Transient transaminase elevation	1a	2 (5.1%)	1 (2.9%)
Syncope	2	1 (2.6%)	0
Biliary complication with sepsis	6	1 (2.6%)	0
Hepatic decompensation	6	1 (2.6%)	0

## Data Availability

An anonymized patient-level dataset is provided in the Supplementary Materials.

## Supplementary Materials

The following supporting information can be downloaded at https://www.mdpi.com/article/10.3390/cancers18132039/s1, Dataset S1: Anonymized clinical and survival dataset of the study cohort. Table S1: Patient- and lesion-level dosimetric parameters according to isotope platform. Values are presented as median (interquartile range).

## Abbreviations

The following abbreviations are used in this manuscript:

TARE	Transarterial radioembolization
HCC	Hepatocellular carcinoma
Ho-166	Holmium-166
Y-90	Yttrium-90
Tc-99m MAA	Technetium-99m macroaggregated albumin
OS	Overall survival
PFS	Progression-free survival
HR	Hazard ratio
CI	Confidence interval
BCLC	Barcelona Clinic Liver Cancer
TACE	Transarterial chemoembolization
PVTT	Portal vein tumor thrombosis
ECOG	Eastern Cooperative Oncology Group
CBCT	Cone-beam computed tomography
SPECT/CT	Single-photon emission computed tomography combined with computed tomography
PET/CT	Positron emission tomography combined with computed tomography
MRI	Magnetic resonance imaging
mRECIST	Modified Response Evaluation Criteria in Solid Tumors
ORR	Objective response rate
CR	Complete response
PR	Partial response
CRR	Complete response rate
DoR	Duration of response
SOD	Sum of diameters
ARLD	Alcohol-related liver disease
MASLD	Metabolic dysfunction-associated steatotic liver disease
SD	Standard deviation
IQR	Interquartile range
CIRSE	Cardiovascular and Interventional Radiological Society of Europe
