# Supplementary material for: Survival Outcomes Following Yttrium-90 and Holmium-166 Transarterial Radioembolization for Hepatocellular Carcinoma
Source: Cancers (Basel). 2026 Jun 24;18(13):2039. doi: 10.3390/cancers18132039 (PMC13360259; doi:10.3390/cancers18132039)
Supplement: Supplementary file 1 [file cancers-18-02039-s001.zip › Table S1.pdf]

Table S1. Patient- and lesion-level dosimetric parameters according to isotope platform. Values are presented as median (interquartile range).

Variable	Yttrium-90 (n=38)	Holmium-166 (n=33)[1]	p-value[2]
**Patient-level dosimetry**			
Administered activity, GBq	2.4 (1.9–3.8)	5.0 (3.5–7.5)	-
Perfused tissue absorbed dose, Gy	235.5 (144.2–330.2)	102.0 (76.0–153.0)	**<0.001**
Whole liver normal tissue absorbed dose, Gy	36.8 (28.3–62.4)	29.0 (19.0–35.0)	**0.003**
**Lesion-level dosimetry**			
Lesions, n	49	40	-
Perfused tumor absorbed dose, Gy	546.6 (393.5–855.1)	205.5 (143.5–319.3)	-

[1] Dosimetric data were available for 71 of 73 patients (38/39 Y-90 and 33/34 Ho-166 patients).

[2] Administered activity was reported descriptively and was not formally compared between isotope platforms. Tumor absorbed dose values are reported at the lesion level; formal statistical comparisons were not performed because patients with multifocal disease could contribute more than one observation.
